# Biomechanics and finite element analysis of a novel plate designed for posterolateral tibial plateau fractures via the anterolateral approach

**DOI:** 10.1038/s41598-023-47575-x

**Published:** 2023-11-17

**Authors:** CongMing Zhang, HuanAn Bai, Teng Ma, Lu Liu, Zhong Li, Kun Zhang, Qiang Huang, Qian Wang

**Affiliations:** Department of Orthopedics, Hong Hui Hospital, Xi’an Jiaotong University, Xi’an, 710054 Shaanxi China

**Keywords:** Trauma, Bone, Bone quality and biomechanics

## Abstract

Surgical management of posterolateral tibial plateau (PLTP) fractures is challenging. One reason for this challenge is the lack of suitable internal fixation devices. Our aim was to introduce a novel plate via the anterolateral approach for managing PLTP fractures. The biomechanical testing and finite element analysis (FEA) were performed. PLTP fracture models were created using synthetic tibias (n = 10 within each group). These models were randomly assigned to three groups (groups A-C) and fixed with the lateral locking plate, the posterior buttress plate, and the novel plate, respectively. The vertical displacement of the posterolateral fragments was evaluated using biomechanical testing and FEA under axial loads of 250 N, 500 N, and 750 N. We also evaluated the stress distribution and maximum stress of each fracture model using FEA. Biomechanically, under the same loads of 250 N, 500 N, or 750 N, the vertical displacement was significantly different among the three fixation groups (*p* ≤ 0.001). FEA data indicated that the maximum displacement from group A to C was 3.58 mm, 3.23 mm, and 2.78 mm at 750 N, respectively. The maximum stress from group A to C was 220.88 MPa, 194.63 MPa, and 156.77 MPa in implants, and 62.02 MPa, 77.71 MPa, and 54.15 MPa in bones at 750 N, respectively. The general trends at 250 N and 500 N were consistent with those at 750 N. Based on our biomechanical and FEA results, the novel plate could be a good option for treating PLTP fractures. The novel plate showed stable and reliable features, indicating its suitability for further clinical application.

## Introduction

With the wide application of computed tomography (CT) in orthopedic trauma, the treatment of posterolateral tibial plateau (PLTP) fractures has become a hotspot in recent years. Previous studies have shown that PLTP fractures account for about 8–15% of all tibial plateau fractures, and the posterolateral column is involved in approximately 54.3% of all lateral tibial plateau fractures^1^. The posterolateral corner of the tibial plateau plays an irreplaceable role in the stability of knee joint flexion. Posterolateral tibial plateau fractures are caused by valgus stress and axial compression stress during knee flexion, which significantly exceed the conventional loads^2^. As this type of fracture belongs to an intra-articular fracture and its stability is poor, most surgeons prefer anatomical reduction and rigid fixation^3^. However, surgical management of PLTP fractures is challenging due to the unique anatomical structures of the posterolateral corner, which is surrounded by the fibular collateral ligament, the fibular head, the common peroneal nerve, and important neurovascular bundles in the popliteal fossa.

Currently, there is no consensus on the optimal surgical approaches and implants for PLTP fractures. Several reports have indicated that the posterior buttress plate can be inserted via several posterior approaches^4,5^. This rigid fixation method can prevent fixation failure and collapse of PLTP fragments. However, due to the abundance of posterior muscle tissues, it is difficult to expose the fracture fragments. Moreover, the posterior approach may result in iatrogenic injuries to the neurovascular bundles in the popliteal fossa. Another commonly used implant for PLTP fractures is the lateral locking plate via the conventional anterolateral approach^6,7^. The anterolateral approach has a lower risk of injuries to the important anatomical structures of the posterolateral corner. However, the screws are inserted from the lateral surface, which lacks effective support for the PLTP fragments. Some studies have reported that the fixation strength of the lateral locking plate is weaker than that of the posterior buttress plate^8^. Surgical failure of PLTP fractures managed using the lateral locking plate is not uncommon in clinical practice^7^. Other scholars have tried modified implants for treating these fractures, such as a new rotational support plate^9,10^. This plate can be inserted via the anterolateral approach and provide support for the PLTP fragments. However, since the fracture fragment is not fixed by screws, fracture displacement frequently occurs after the operation.

**Figure 1:**
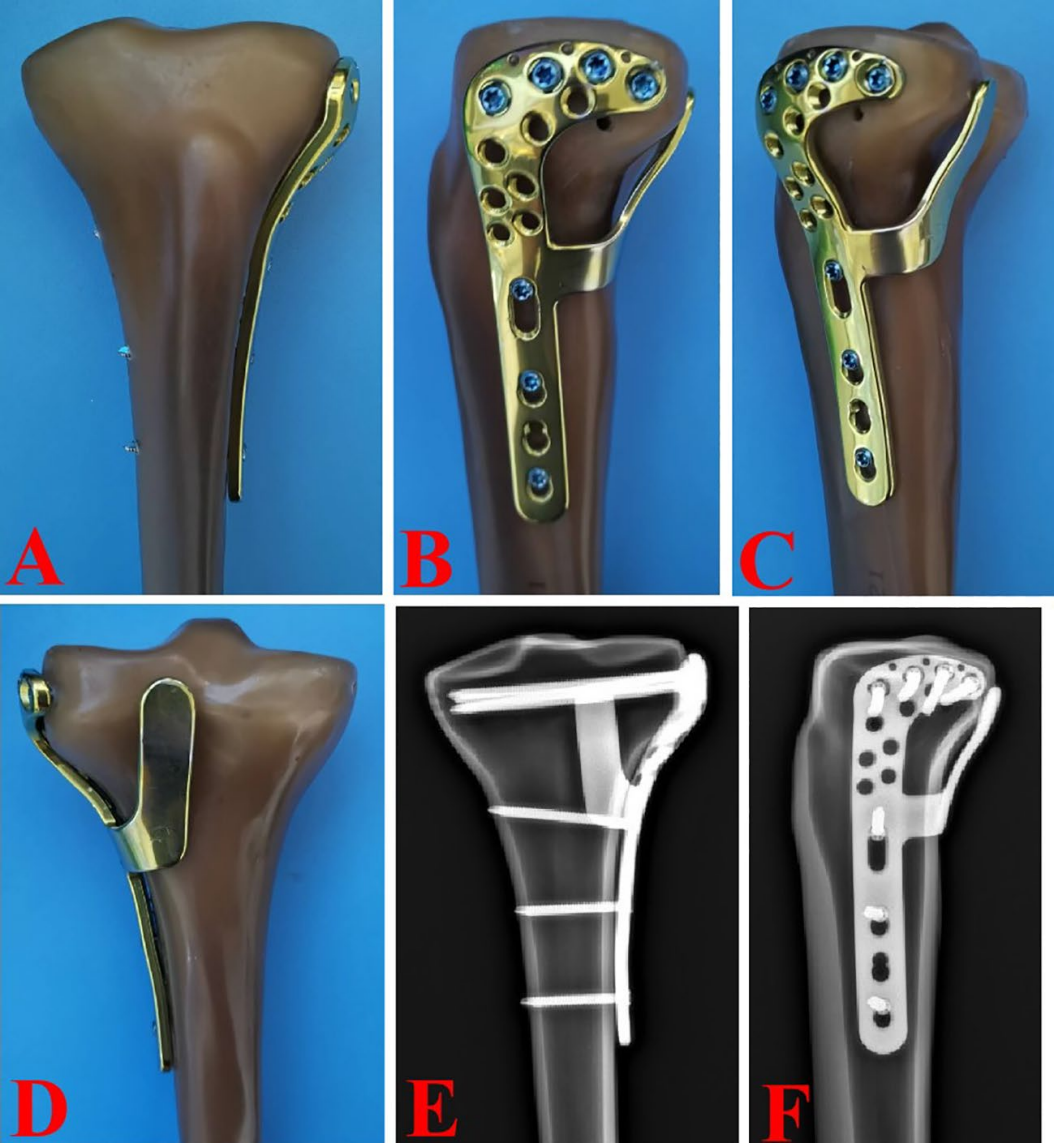
Mock-ups of the novel plate in synthetic bones. (**A**–**D**) Anterior, lateral, posterolateral and posterior views of the novel plate. (**E**,**F**) Anteroposterior and lateral view of the X-ray images.

The available fixation methods for PLTP fractures are relatively limited. Additionally, ensuring the safety of the surgical approach and stability of the implants simultaneously remains a challenge. Therefore, there is an urgent need to treat PLTP fractures by inserting appropriate implants via a relatively simple approach. Based on these considerations, we have designed a novel plate for patients with PLTP fractures. As shown in Fig. 1, this plate features a two-arm design and is predestined to be inserted via the traditional anterolateral approach. The posterior buttress arm is meant to be positioned via the proximal tibiofibular space, while the anterolateral inverted L-shaped arm can properly fix the PLTP fragment from the space above the fibular head. The four holes at the proximal part of the anterolateral arm are universal holes that allow for effective fixation of the posterolateral fragment. The posterior oblique arm can pass through the upper tibiofibular space and buttress the PLTP fragment from the posterior side. Moreover, when the PLTP fragment is comminuted or combined with collapse, the two-arm design of our novel plate can simultaneously support and effectively fix the PLTP fragment. Our novel plate is anatomically designed and adheres well to the PLTP fragment.

In this study, we created a PLTP fracture model using artificial bone models. We compared the biomechanical effects of the novel plate with two conventional implants for treating PLTP fractures. We also performed finite element analysis (FEA) to explore the efficacy of the novel plate. Our hypothesis was that the novel plate would provide adequate stability and exhibit the best biomechanical strength and FEA performance.

## Materials and methods

The institutional review board approval has been obtained from the ethics committee of Xi’an Hong Hui Hospital. Written informed consent was obtained from the participants, and all methods were conducted in accordance with relevant guidelines and regulations. Synthetic left tibias (n = 30) were used to create PLTP fracture models. Three types of implants, including 3.5-mm novel plates (Titanium alloy, Naton Medical Instrument Co., Ltd., Tianjin, China), 3.5-mm posterior buttress plates (Titanium alloy, Naton Medical Instrument Co., Ltd., Tianjin, China), and 3.5-mm lateral locking plates (Titanium alloy, Naton Medical Instrument Co., Ltd., Tianjin, China), were inserted to fix these fracture models. The anatomical parameters of the 3.5-mm novel plate were based on Chinese populations. Biomechanical performance of PLTP fracture models was assessed using the Electroforce 3520-AT electronic universal material testing machine.

### Fracture models and groups

**Figure 2:**
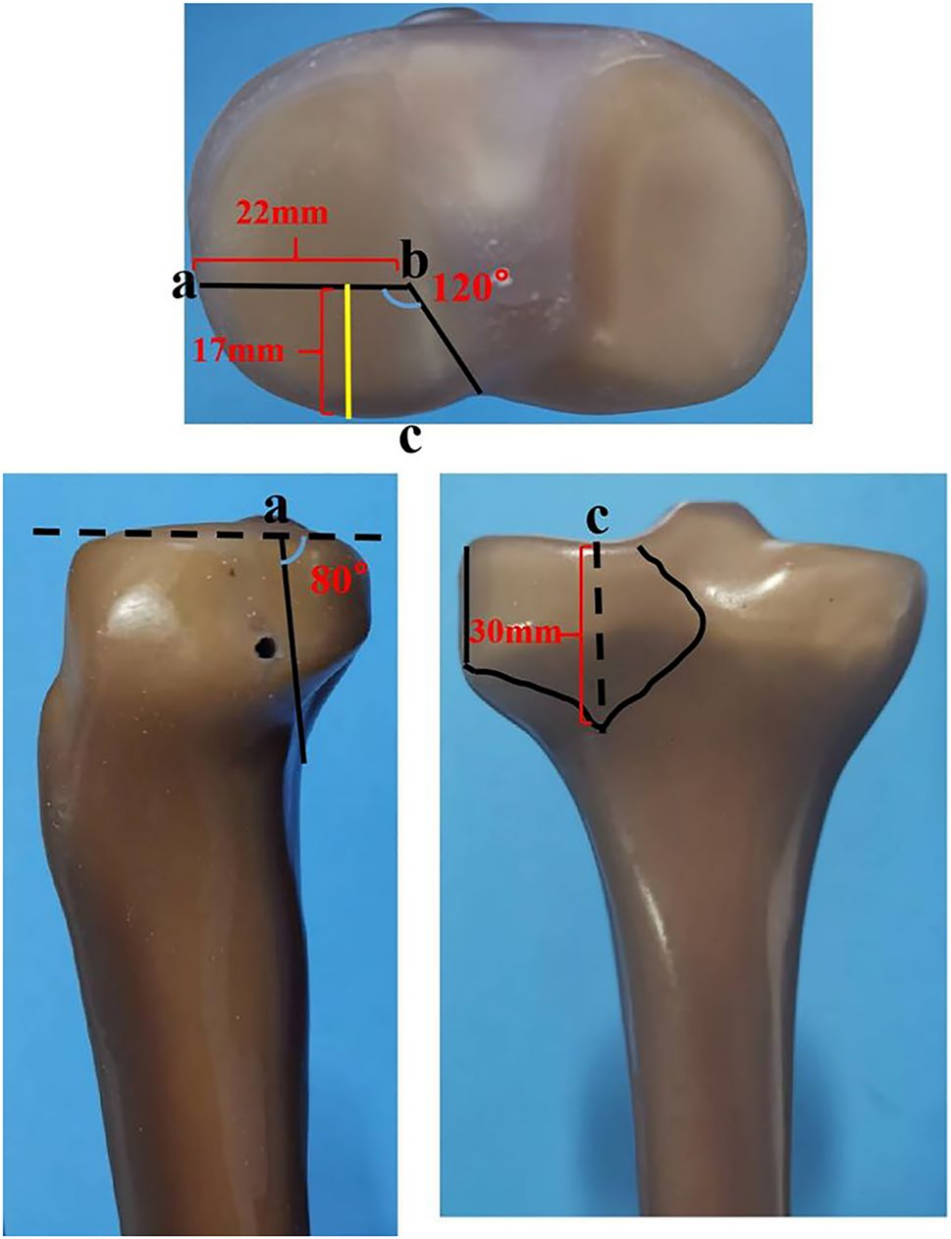
Model of the posterolateral tibial plateau fracture.

**Figure 3:**
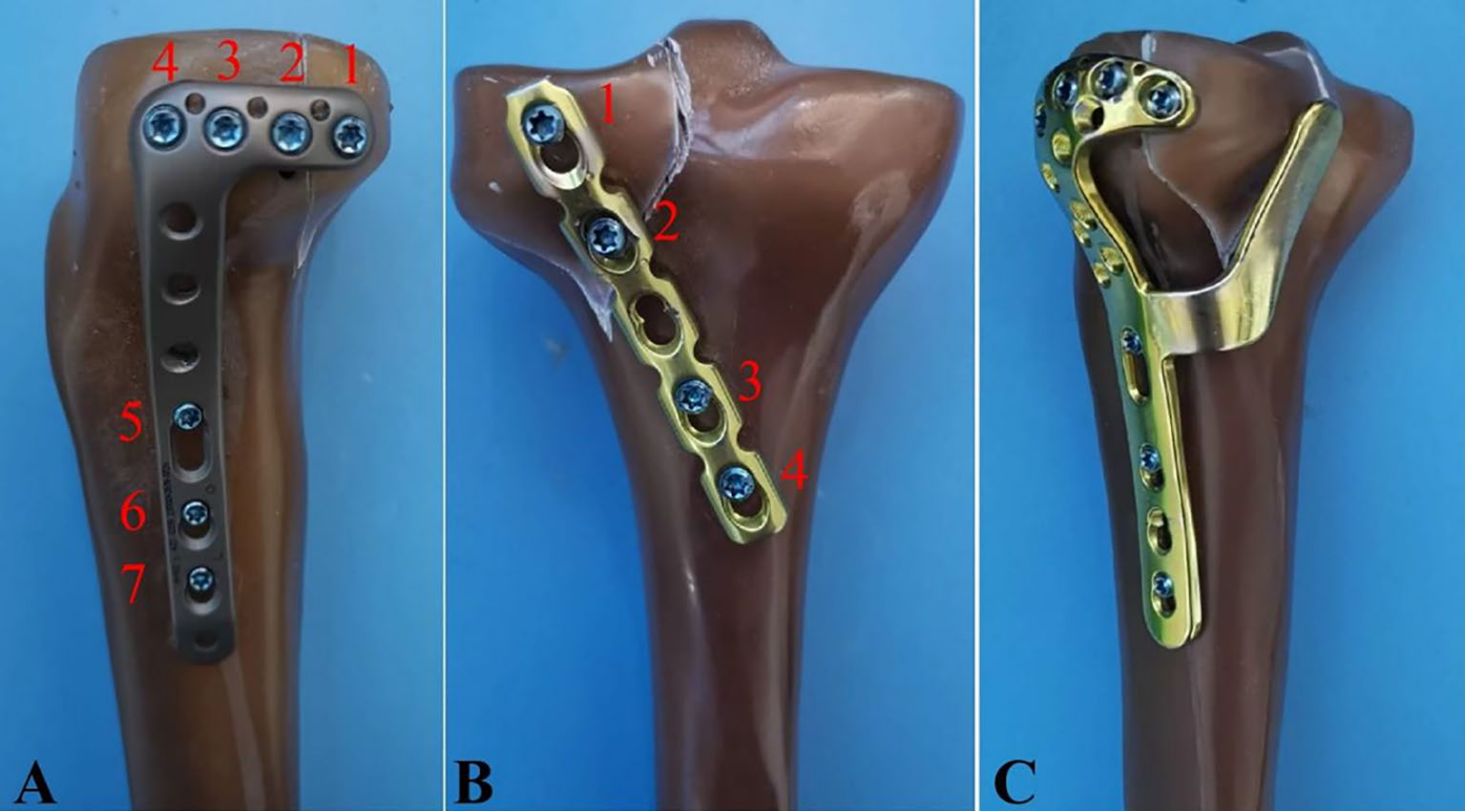
PLTP fracture models with three different implants in biomechanical tests. (**A**) Fixation of the lateral locking plate in the PLTP fracture. (**B**) Fixation of the posterior buttress plate in the PLTP fracture. (**C**) Fixation of the novel plate in the PLTP fracture. *PLTP* posterolateral tibial plateau. Red numbers stand for screw identifiers.

**Figure 4:**
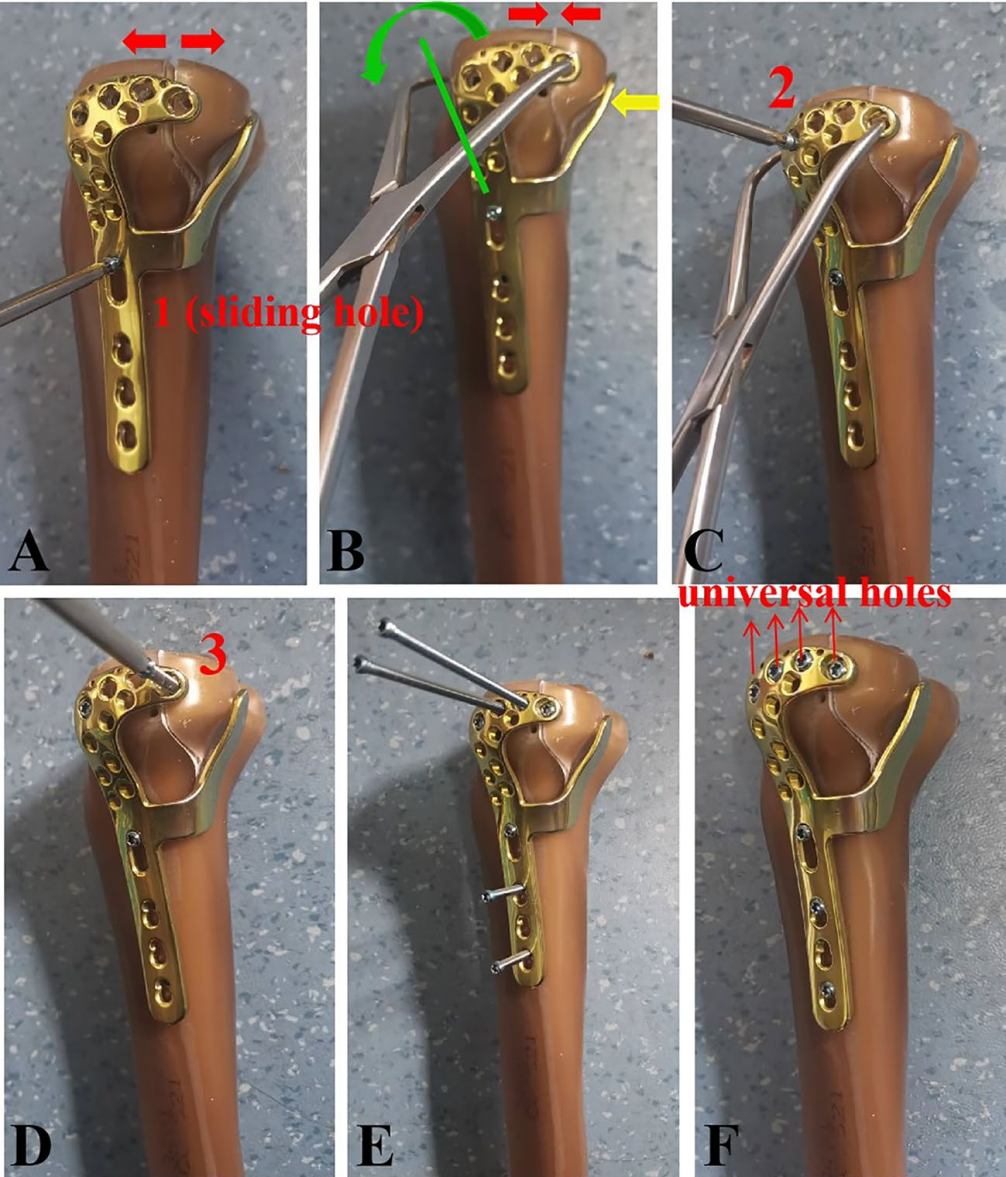
Fixation procedures of the novel plate in the PLTP fracture. (**A**) The first screw is inserted through the sliding hole located at the junction of two arms. At this point, the fracture gap still exists. (**B**) The proximal part of the novel plate is slightly pulled forward by a resetting forceps. Then, the posterior buttress arm could compact the PLTP fragment tightly, and the fracture gap will decrease. (**C**) The second screw is inserted in the anterolateral corner of the horizontal arm. (**D**) The third screw is inserted through the universal hole at the posterior part of the horizontal arm to fix the PLTP fragment. (**E**) The remaining holes are fixed by screws in turn. (**F**) The PLTP fragment is well fixed by the novel plate. *PLTP* posterolateral tibial plateau.

PLTP fractures were simulated according to a previous study^9^ by means of osteotomies using a thin saw blade (Fig. 2). All geometric measurements and preparations were performed by the same surgeon. Prior to plate fixation, the fragments were anatomically reduced and provisionally fixed with reduction clamps. Once the fracture was created, the tibia models were divided into three groups for fixation. Group A was fixed with a 3.5-mm lateral anatomic locking plate (Fig. 3A). Seven 3.5-mm locking screws were inserted from the lateral side of the tibia to the medial side. Screw 1 captured the posterolateral fragment while Screws 2, 3 and 4 captured the tibia plateau. After inserting Screws 1, 2, 3, and 4 parallel to the articular surface, Screws 5, 6, and 7 were inserted in turn. Group B was fixed with a posterior five-hole straight buttress plate (Fig. 3B). Four 3.5-mm locking screws were inserted from the posterior side of the tibia to the anterior side. The plate was properly prebent. Screws 1 and 2 captured the posterolateral fragment, and Screws 3 and 4 captured the tibia plateau. Group C was fixed with the novel plate (Fig. 3C). As shown in Fig. 4A, the first screw was inserted through the sliding hole at the junction of two arms, followed by slight pulling of the proximal part of the plate forward using resetting forceps (Fig. 4B). One end of the resetting forceps was held at the proximal part of the novel plate, and the other end was held at the anterior side of the tibial plateau. This allowed the posterior buttress arm to compact the PLTP fragment tightly. The second screw was then inserted in the anterolateral corner of the horizontal arm (Fig. 4C), significantly reducing the fracture gap. The third screw was inserted through the universal hole at the posterior part of the horizontal arm to fix the PLTP fragment (Fig. 4D). Finally, the remaining screws were inserted and tightened with consistent torque (Fig. 4E,F).

### Biomechanical testing

**Figure 5:**
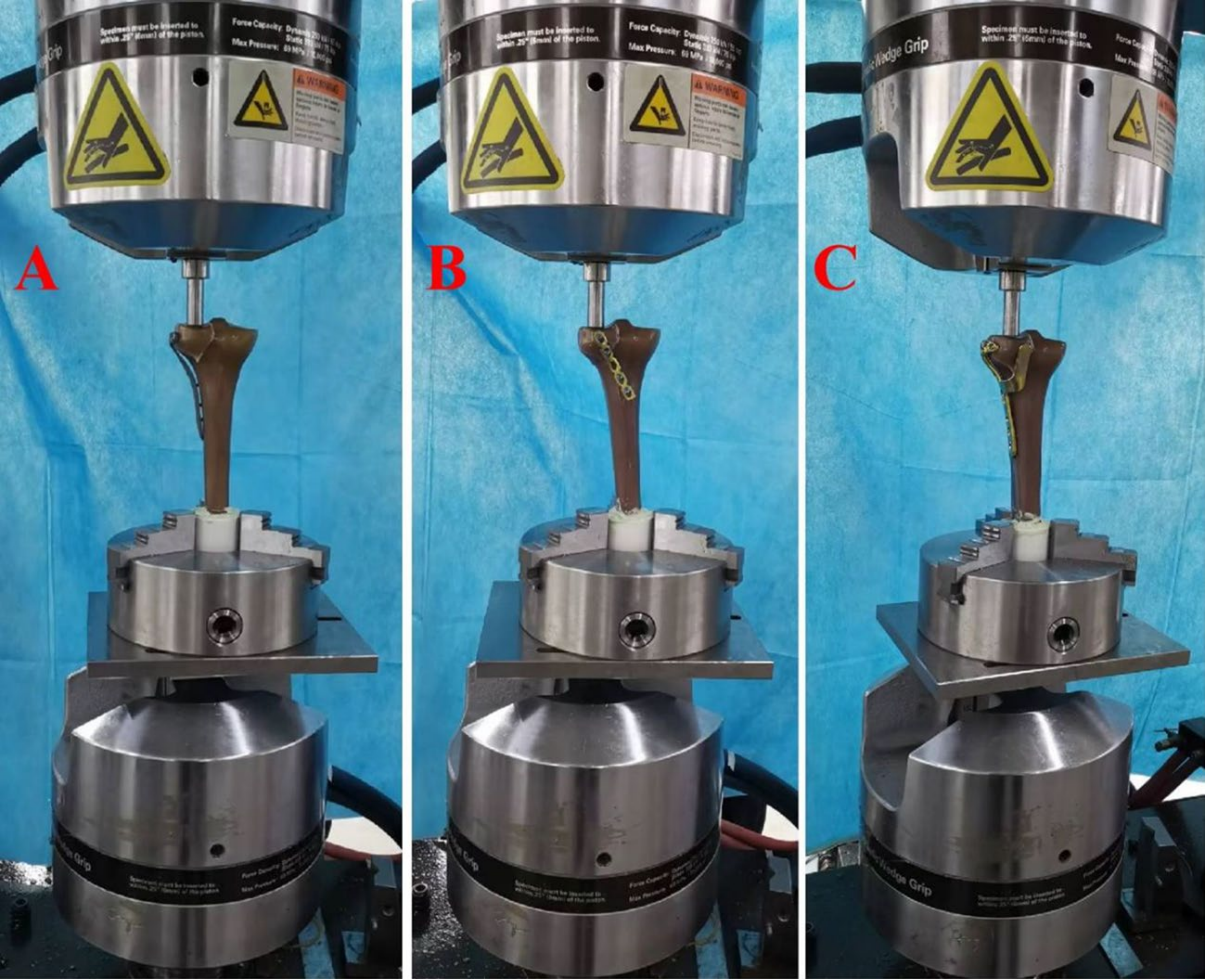
Positioning of three different tibial models within the biomechanical machine. (**A**) Fixation of the lateral locking plate in the PLTP fracture. (**B**) Fixation of the posterior buttress plate in the PLTP fracture. (**C**) Fixation of the novel plate in the PLTP fracture. *PLTP* posterolateral tibial plateau.

Each assembled PLTP fracture model was fixed vertically on a material testing machine (Fig. 5). Vertical loads were applied to the upper surface of the PLTP fragment using a custom cylindrical indenter with a diameter of 15 mm. Previous studies have indicated that under normal gait, the biomechanical loads applied to the knee are approximately two to three times of normal body weight^11^. The lateral tibial plateau bears about 45% of the loads, while the medial tibial plateau bears approximately 55%^12^. To simulate the loads applied to the posterolateral column during normal gait, axial peak loads of 250 N, 500 N, and 750 N were set for the synthetic fracture models. The biomechanical testing was aimed to simulate the static phase for three fixation models in the biomechanical testing machine. After the PLTP fracture model was assembled in the machine, gradually increased axial loads were applied to each model at a speed of 10 N/s. Vertical displacement of the PLTP fragment was then recorded and the displacement values were exported to a specific software. The load–displacement curves were generated for each model. Fixation failure was defined as vertical displacement of the PLTP fragment exceeding 3 mm^13^. The maximum peak loads were set at 750 N or the loads at a displacement of 3 mm for the PLTP fragment. Vertical displacements at these loads were used to evaluate the efficacy of the internal devices.

### Finite element analysis

A healthy male volunteer, aged 40, with no history of knee joint or systemic diseases, was recruited for the study. A 64-row spiral CT scanner was used to perform a CT scan with a layer thickness of 0.625 mm from the knee to the ankle. The resulting CT images were stored in DICOM format and imported into the interactive medical image control system (Mimics 19.0, Materialise, Leuven, Belgium). Based on grayscale values and region segmentation, a 3D tibial model was constructed. Subsequently, the 3D images were divided into surface meshes using Mimics’ Magics 9.9 rigid-lattice division program, while optimizing the 3D models and creating the corresponding surface meshes. These surface mesh models were then converted into volume mesh models using a Mesh tool (ANSYS, Inc., Canonsburg, PA, USA). The volume mesh models were re-imported into Mimics to obtain material properties. All three models featured homogeneous and linear isotropic material properties and were meshed using tetrahedral elements. To ensure model reliability, a convergence study was conducted, which confirmed that field variables such as strain energy and displacement remained within 5% for both types of elements, and there were no points of maximum stress.

**Figure 6:**
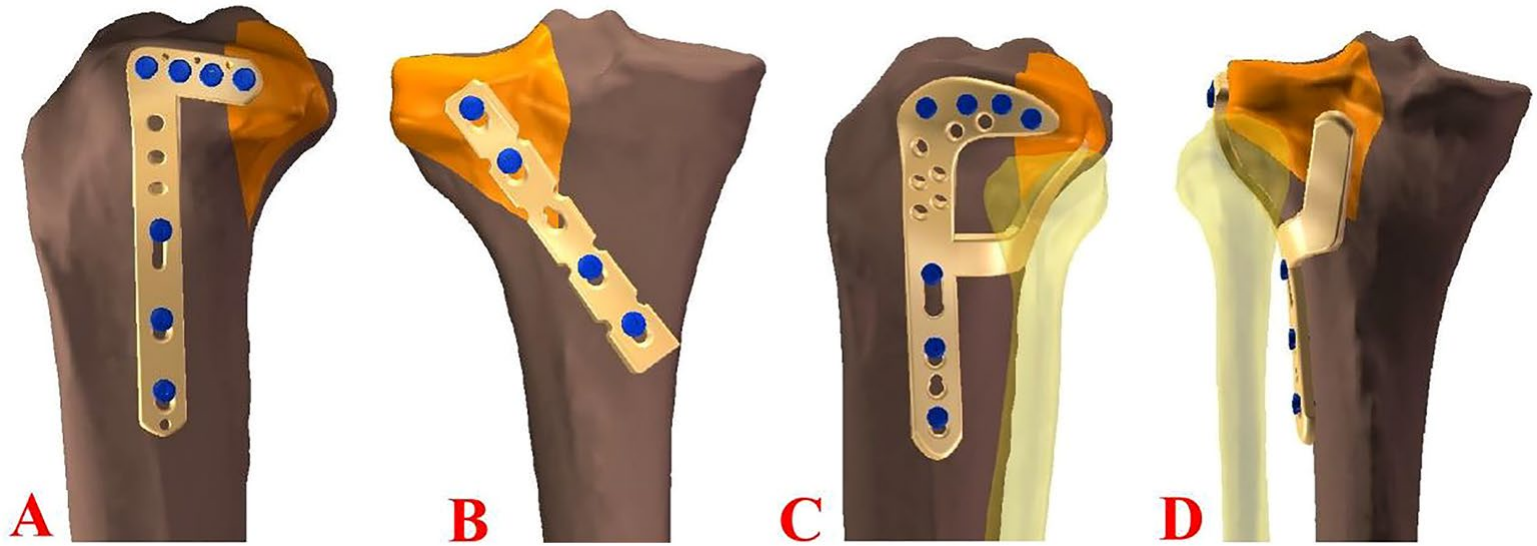
Three different implants after assembly of the finite element model. (**A**) Fixation of the lateral locking plate in the PLTP fracture. (**B**) Fixation of the posterior buttress plate in the PLTP fracture. (**C**,**D**) Fixation of the novel plate in the PLTP fracture. *PLTP* posterolateral tibial plateau.

The Young’s modulus for cortical bones, cancellous bones, plates, and screws were defined as 14,000 MPa, 700 MPa, and 110,000 MPa, respectively, based on previous literature^14,15^. The 3D models of plates and screws were created using manufacturer-provided specifications and computer-aided design (CAD) software. Frictional contact was defined for all interfaces, including those between different fracture fragments, between the fracture fragment and the plate, and between the plate and screws. A friction coefficient of 0.4 was selected based on previous literature^16^. Figure 6 illustrates the creation of PLTP fracture models by incising fracture lines, followed by the assembly of plates and screws using specific software. The fixation methods employed in this process were consistent with those used in biomechanical testing. Table 1 provides the quantities of elements and nodes for the three different fixation models.

**Table 1:** Number of nodes and elements for the three different models.

Model	Nodes	Elements
Group A	952,427	633,813
Group B	865,671	583,283
Group C	991,035	657,600

*Group A* the lateral locking plate, *Group B* a posterior buttress plate, *Group C* the novel plate.

The inferior third of the tibia shaft was fixed in all degrees of freedom. The PLTP fragments were compressed by the axial loads of 250 N, 500 N, and 750 N. The vertical loads were applied to the upper surface of the PLTP fragments using a simulated cylindrical indenter with a 15 mm diameter. ANSYS Mechanical APDL 19.0 (ANSYS, Inc., USA) was employed to manage all fracture models. FEA was conducted to simulate static testing for the three different fixation devices. The maximum vertical displacement of each fracture model under various axial loads was analyzed and recorded. Additionally, the von Mises stress distribution and maximum von Mises stress of each model were also examined and documented.

### Statistical analysis

SPSS 22.0 (SPSS, Inc., Chicago, IL, USA) was used to statistically analyze data derived from experimental biomechanical testing. Student's t-tests were used to compare measurement data. Two-way Analysis of Variance with Repeated Measures was performed to analyze the differences in vertical displacement and axial loads of the PLTP fragment among the three groups. Fisher’s post hoc test and least-significant difference criterion were used for multiple group comparisons. Statistical significance was defined as *p* < 0.05.

### Consent to participate/consent to publish

The participant has signed the informed consent and provided the consent to publish and report individual clinical data.

## Results

### Biomechanical testing

**Figure 7:**
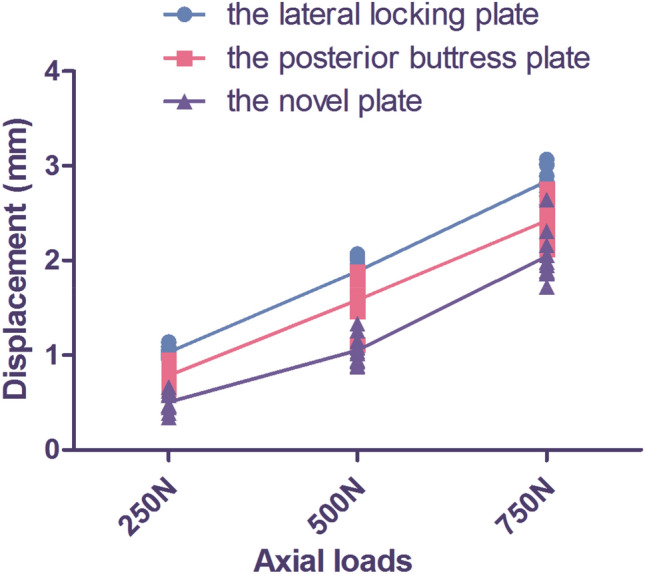
Vertical displacement under three different axial loads of the three different fixation groups. Axial loads included 250 N, 500 N, and 750 N. The three fixation groups included the lateral locking plate group, the posterior buttress plate group, and the novel plate group. The horizontal axis represents the different axial loads. The vertical axis represents vertical displacement.

The vertical displacement under three different axial loads is presented in Fig. 7. Significant differences in vertical displacement were observed among the three different fixation groups for the same loads of 250 N, 500 N, or 750 N (*p* ≤ 0.001). Within each group, the vertical displacement was also significantly different among the three different axial loads (*p* ≤ 0.001). Notably, as the axial loads increased, there was an increasing trend in vertical displacement.

Regarding the failure loads of the models, the three different fixation groups were Group A–B–C, ranking from the lowest to the highest. The novel plate group (Group C) exhibited a significantly higher load-bearing capacity compared to the lateral locking plate group (Group A) (*p* ≤ 0.001) and the posterior buttress plate group (Group B) (*p* ≤ 0.001). The specific failure loads were as follows: 811.72 ± 42.94 N for the lateral locking plate group (Group A), 910.43 ± 49.44 N for the posterior buttress plate group (Group B), and 1045.01 ± 50.47 N for the novel plate group (Group C).

### Finite element analysis

**Figure 8:**
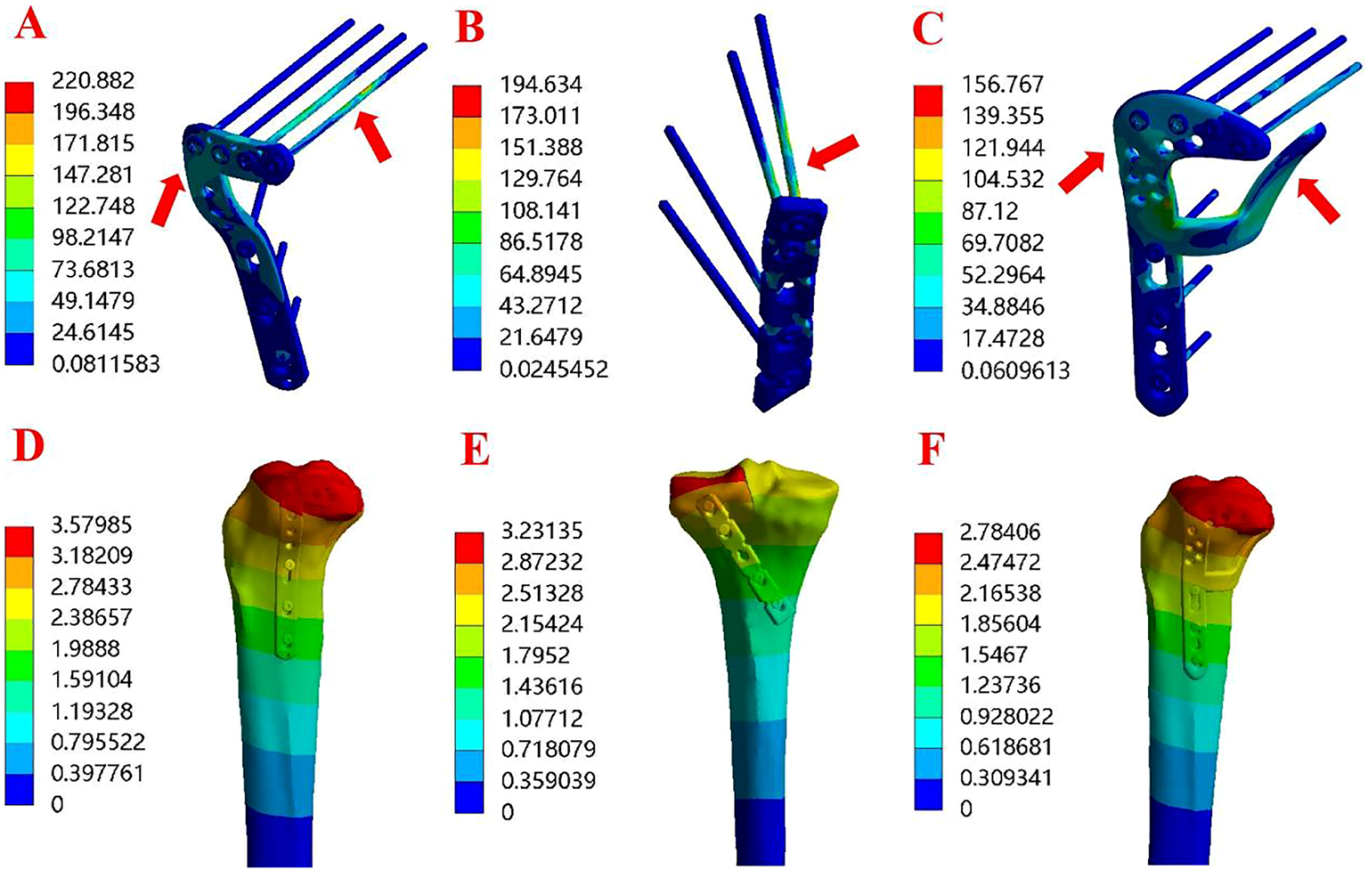
Stress distribution diagram and displacement field of the three finite element models under axial loads of 750 N. (**A**) Stress distribution of model A in the PLTP fracture. (**B**) Stress distribution of model B in the PLTP fracture. (**C**) Stress distribution of model C in the PLTP fracture. (**D**) Displacement field of model A in the PLTP fracture. (**E**) Displacement field of model B in the PLTP fracture. (**F**) Displacement field of model C in the PLTP fracture. The areas indicated by the red arrows are the stress concentration areas. *PLTP* posterolateral tibial plateau.

As depicted in Fig. 8A, the von Mises stress distribution of the lateral locking plate (Group A) was primarily concentrated on the two locking screws at the posterior side of the horizontal arm and the corner of the L-shaped plate. In Fig. 8B, the von Mises stress distribution of the posterior buttress plate (Group B) concentrated on the proximal two screws. For the novel plate (Group C), shown in Fig. 8C, the von Mises stress distribution was concentrated on the posterior arm and the junction corner of the anterolateral arm. Notably, the von Mises stress increased along with the increase of axial loads for all three different fixation devices. Under an axial load of 750 N, the maximum von Mises stress ranged from 220.88 MPa (Group A) to 194.63 MPa (Group B) and 156.77 MPa (Group C). The von Mises stress distribution was consistent with the load increase from 250 to 500 N. Table 2 summarizes the von Mises stress values for the three different fixation devices under the specified loads. Furthermore, the maximum von Mises stress applied to the bones ranged from 62.02 MPa (Group A) to 77.71 MPa (Group B) and 54.15 MPa (Group C) under a load of 750 N (Table 2). Notably, the novel plate exhibited a lower maximum von Mises stress on the bones compared to the lateral locking plate and the posterior buttress plate for PLTP fractures.

**Table 2:** Maximum displacement, maximum von Mises stress in implants and bones of the finite element models of PLTP fracture.

Group	Max displacement (mm)			Max von Mises stress in implants (MPa)			Max von Mises stress in bones (MPa)		
	250 N	500 N	750 N	250 N	500 N	750 N	250 N	500 N	750 N
A	1.17	2.34	3.58	88.41	157.01	220.88	20.67	41.34	62.02
B	0.96	1.98	3.23	63.77	117.65	194.63	20.96	40.01	77.71
C	0.74	1.48	2.78	52.93	106.71	156.77	12.91	35.69	54.15

*PLTP* posterolateral tibial plateau.

The vertical displacements of each group under 250 N, 500 N, and 750 N using FEA are summarized in Table 2. Figure 8D–F shows the displacement of three fixation models under an axial load of 750 N. From the highest to the lowest value of this index, the three fixation models were sorted as follows: Group A–B–C. The overall trends in vertical displacement were consistent among the three different fixation devices as the axial loads increased from 250 to 750 N. Moreover, the vertical displacement of the PLTP fragment in each model gradually increased with the axial loads ranging from 250 to 750 N.

## Discussion

Posterolateral tibial plateau (PLTP) fractures have gained significant attention recently. The results of the biomechanical testing indicated that our novel plate showed better biomechanical efficacy for PLTP fractures compared to the lateral locking plate and the posterior buttress plate. Finite element analysis also demonstrated that the novel plate exhibited minimal displacement of PLTP fragments, aligning with the findings from the biomechanical testing. Additionally, the maximum von Mises stress and stress distribution were evaluated using a 3D model in the FEA. The novel plate showed lower maximum von Mises stress compared to the lateral locking plate and the posterior buttress plate. The design of the posterior buttress arm effectively disperses stress from the anterolateral arm, thus preventing stress concentration near the fracture line. It indicates that the novel plate may have a low risk of biomechanical failure, and the two-arm design is feasible.

In the lateral locking plate group, although four screws were inserted parallel to the articular surface, only one screw was able to capture the PLTP fragment. As a result, the PLTP fragment is prone to displacement, and stress concentration occurs at Screw 1. This makes the lateral locking plate group more susceptible to fixation failure compared to the other groups. In the posterior buttress plate group, although two screws capture the PLTP fragment, Screw 2 only captures the tip of the wedge-shaped PLTP fragment, limiting its fixing effect. This may explain why the biomechanical stability of the posterior buttress plate is inferior to that of the novel plate.

Based on our data, the novel plate offered several advantages. Firstly, it could be inserted through the anterolateral approach, preserving the integrity of the posterolateral complex and avoiding damage to important vessels and nerves. The insertion process was relatively simple, reducing blood loss and shortening operation time. Secondly, the novel plate demonstrated good biomechanical characteristics, and its two-arm design enabled three-dimensional fixation of the PLTP fragment. Thirdly, when combined with the anterolateral column fragment, this plate could fix fracture fragments involving both the anterolateral and posterolateral columns simultaneously. Lastly, the removal of this plate was relatively simple once the PLTP fracture has healed.

Although several surgical approaches and implant designs have been developed for PLTP fractures, a consensus on the optimal approach and implant design has yet to be reached. Various approaches and fixation devices have been explored by scholars. Yu et al.^17^ utilized the fibular head osteotomy approach in the treatment of 82 cases involving lateral and PLTP fractures. Follow-up results revealed chronic pain in six cases, tibial plateau height loss in three cases, and lateral instability of the knee joint in three cases. This approach provided full exposure of the fracture fragment, facilitating reduction and fixation of the PLTP fragment. However, it was important to note that this approach carried the risk of common peroneal nerve injury, fibular nonunion, and upper tibiofibular joint injury. Other experts have focused on different posterior approaches for PLTP fractures. Carlson et al.^18^ employed the posterolateral “S”-shaped incision approach, located above the biceps femoris. During operation, the common peroneal nerve behind the biceps femoris should be exposed and pulled out for protection. Tao et al.^5^ used a modified inverted “L” incision to treat 11 cases of PLTP fractures. It is crucial to fully expose the space between the biceps femoris and gastrocnemius muscle, ensuring the release and protection of the common peroneal nerve during the operation. Liu et al.^19^ achieved favorable outcomes using the modified posterolateral straight incision approach without exposing the common peroneal nerve in the treatment of nine cases of simple PLTP fractures. Similarly, Frosch et al.^20^ proposed an extended posterolateral incision technique, reducing and fixing anterolateral and PLTP fractures through the respective muscle spaces. However, it is important to acknowledge that the posterolateral anatomy of the knee joint is intricate, with proximity to important vessels and nerves, making the surgery relatively challenging. Although an anterolateral approach provides easy surgical exposure to the PLTP, there are still several limitations, including insufficient exposure and inadequate reduction of the fracture fragment.

Customization of internal fixation devices is essential for different surgical approaches in treating PLTP fractures. Typically, a 3.5-mm lateral anatomical locking plate is selected for the anterolateral approach, while a 3.5-mm distal radius T-shaped or reconstruction plate is chosen for posterior approaches, with final shaping occurring during the operation^5,8,19,21^. Biomechanical experiments have consistently demonstrated the significant biomechanical advantages of posterior buttress plates^8,22^ in effectively preventing reduction loss due to shear stress on the fracture fragment. However, it is important to limit the length of the posterior plate to approximately 5 cm to avoid damage to the anterior tibial artery. Consequently, although effective approaches and internal fixation devices are available, they all possess certain limitations. Currently, there is no consensus regarding relatively safe approaches and suitable internal fixation devices for PLTP fractures. To address these challenges, we have developed a novel two-armed plate. Our biomechanical testing and FEA results demonstrated that this novel plate exhibited superior biomechanical stability compared to both the lateral locking plate and the posterior buttress plate. Moreover, this plate could be inserted through the anterolateral incision, potentially reducing trauma and the occurrence of complications.

There are several limitations of this study. It's important to note that although cadaver bones are considered optimal test materials, we used synthetic tibias in our study due to their uniformity, consistent material properties, geometry, and mechanical characteristics^23^. The synthetic tibia models allowed for reliable assessment and comparison of the three different plates; however, it does not fully replicate the complexities of a clinical situation. Moreover, factors such as soft tissues, ligaments, meniscus, and muscle tissues were not taken into account in our biomechanical testing and FEA, which only simulated a simplified scenario^24,25^. Additionally, the shape, size and comminution of the PLTP fragment may affect the selection of implants. Our study only focused on a specific type of common posterolateral fracture. Finally, biomechanical testing of this study was not in-depth enough. There were some flaws in the methodology regarding biomechanical testing, such as the omittance of stiffness evaluation and precise motion tracking, and cyclic loading. These deficiencies will be improved in further research.

## Conclusion

In conclusion, our study presented a new option for the management of PLTP fractures. The biomechanical testing and FEA results provided evidence for the reliability of our novel plate. Further clinical series on PLTP fractures will be conducted to strengthen the basis for its future clinical application.

## Data Availability

The datasets analyzed during the current study are available from the corresponding author upon reasonable request.
